# Clustering of health and risk behaviour in immigrant and indigenous Dutch residents aged 19–40 years

**DOI:** 10.1007/s00038-012-0350-4

**Published:** 2012-02-28

**Authors:** Sijmen A. Reijneveld, Maroesjka van Nieuwenhuijzen, Mariska Klein Velderman, Theo W. G. M. Paulussen, Marianne Junger

**Affiliations:** 1 Department of Health Sciences, University Medical Center Groningen, University of Groningen, PO Box 196, 9700 AD Groningen, The Netherlands; 2 TNO (Netherlands Organisation of Applied Scientific Research) Quality of Life, Leiden, The Netherlands; 3 Department of Clinical Child and Family Studies and the EMGO Institute for Health and Care Research, VU University Amsterdam, Amsterdam, The Netherlands; 4 Department of Social Risks and Safety Studies, University of Twente, Enschede, The Netherlands

**Keywords:** Health behaviours, Delinquency, Clustering, Minority groups, Immigration

## Abstract

**Objectives:**

Studies on the co-occurrence, ‘clustering’ of health and other risk behaviours among immigrants from non-industrialised countries lack until now. The aim of this study was to compare this clustering in immigrant and indigenous adults.

**Methods:**

A representative sample (*N* = 2,982; response 71%) of the Dutch population aged 19–40, with 247 respondents from non-industrialized countries (Turkey, Morocco, Surinam, Netherlands Antilles), was asked about health behaviours (alcohol, smoking, drugs, unsafe sex, exercise, nutrition, sleep behaviour, traffic behaviour), and about rule-breaking behaviour and aggression. Data were collected using internet questionnaires, which excluded respondents unable to read Dutch.

**Results:**

Among indigenous adults, health and risk behaviours co-occur in three clusters (alcohol, health-enhancing behaviour, and rule-breaking behaviour), whereas among immigrant groups two clusters were found (alcohol and rule-breaking behaviour/smoking). Differences mostly concerned health-enhancing behaviours such as nutrition, which was not part of any cluster, and physical activity.

**Conclusions:**

This supports an integrated promotion of healthier lifestyles to immigrants who are able to read Dutch. Regarding potentially risky behaviours like alcohol use and rule-breaking behaviours, this could be similar to that for indigenous people.

## Introduction

Health and risk behaviours, such as smoking, poor diet, physical inactivity, excessive alcohol consumption, motor vehicle crashes, risky sexual behaviour, delinquency and illicit drug use, have a major impact on health and mortality (Emberson et al. 2005; Knoops et al. 2004; Meng et al. 1999; Mokdad et al. 2004; Yusuf et al. 2004). The greater the involvement in more risky behaviours, the higher the negative effect on health (Meng et al. 1999; Spencer et al. 2005; Yusuf et al. 2004), both in regard to health behaviours, and to aggression and delinquency (Piquero et al. 2007; Shepherd et al. 2009). These negative effects may cumulate if risky behaviours co-occur in people (Burke et al. 1997; Faeh et al. 2006; Ma et al. 2000; Poortinga 2007; Pronk et al. 2004; Schuit et al. 2002; Wiefferink et al. 2006). Recently, this co-occurrence has been demonstrated for a wide range of health and risk behaviours, with co-occurrence being even more likely for some groups of behaviours, denoted as ‘clusters’. For adults, the clusters were health-enhancing behaviours (like physical exercise and intake of fruit and vegetables), alcohol consumption and delinquent/rule-breaking behaviours (van Nieuwenhuijzen et al. 2009). As yet there is no evidence on differences in this clustering between immigrant groups and the indigenous population.

Immigrants from non-industrialised countries often start in a socioeconomically relatively disadvantaged position when migrating to industrialised countries (International Organisation for Migration 2008; Nielsen and Krasnik 2010; Reijneveld 2010). This also applies to most of the major immigrant groups in the Netherlands (Hosper et al. 2007; Nierkens et al. 2006; Reijneveld 1998a). Major groups come from Turkey and Morocco—migrants who came to the Netherlands as unskilled labourers in the 1960s and 1970s. Other major groups come from Surinam and the Netherlands Antilles, former Dutch colonies. Surinam obtained independence in the 1970s leading to a large migration wave thereafter; the Netherlands Antilles are still connected to the Netherlands. Immigrants who were born outside the Netherlands are called first-generation immigrants; their children who are born in the Netherlands are called second-generation immigrants.

The health behaviours of these immigrant groups differ from the indigenous Dutch population, albeit not always in an unfavourable direction (Cornelisse-Vermaat and van den Brink 2007; Hawkins et al. 2008; Hosper et al. 2007; Nierkens et al. 2006; Reijneveld 1998a). Alcohol consumption and physical activity are lower in all immigrant groups. Smoking prevalence rates vary by group, generation and gender, being higher among Turkish and Surinamese men, for example, especially in the first generation, but much lower among Moroccan women, again more pronounced in the first generation (Hosper et al. 2007; Nierkens et al. 2006; Reijneveld 1998a). In addition, rates of delinquent behaviour have been shown to be higher in all immigrant groups (Blom and Jenissen 2007).

Differences between immigrants and the indigenous population in the clustering of health behaviours and rule-breaking behaviours might be associated with the differences in prevalence rates but evidence on this topic is lacking. Such evidence is sorely needed to determine whether it may be of use to address several immigrant health behaviours simultaneously, in integrated prevention programmes, i.e. programmes that target the joint determinants of several health behaviours, such as effective parenting, or creating a school or work-place environment that supports the acceptability of healthy behaviours among school or work peers. Therefore, the aim of the present study was to examine the clustering of a wide range of health-enhancing and health-endangering behaviours by migration status. For practical reasons, we focused on immigrants who were able to fill out an internet-based questionnaire. We assumed that if differences with the indigenous Dutch group were found, these would be greater in the groups that are not able to fill out such questionnaires, given the strong relationship between language, acculturation and change of health behaviours towards those of the majority population (Hunt et al. 2004; Salant and Lauderdale 2003). Moreover, we focussed on young adults as health behaviours, once established, tend to continue throughout life (Due et al. 2011; Shepherd et al. 2009).

## Methods

### Population

The respondents were a random sample of Dutch residents, stratified by age, sex and educational level of the head of household, and limited to those aged 12–40 years. Overall response was 71% (*N* = 3,423); among immigrants the response rate was 83%. The current analyses are restricted to those aged 19–40 because the number of immigrant adolescents was too low to enable separate analyses, and a previous study shows that they have a different clustering of health behaviours (van Nieuwenhuijzen et al. 2009). Moreover, immigrants from countries other than Turkey, Morocco, Surinam and the Netherlands Antilles were excluded because of their small numbers, which hindered further analyses. The remaining sample comprised 2,943 people, including 247 immigrants. The sample was representative for the Dutch population except that immigrants not able to read Dutch were excluded. This concerns a significant proportion of the Turkish and Moroccan first-generation immigrants, but not of the other groups, Dutch being the official language of Surinam and the Netherlands Antilles and education having full-population coverage in those two countries. Further details on the data collection have been reported elsewhere (van Nieuwenhuijzen et al. 2009). Ethical approval was gained from the ethical committee of the Faculty of Social Sciences, Utrecht University, The Netherlands.

### Procedure and measures

Respondents were asked to fill out an internet-based questionnaire on health behaviours, rule-breaking behaviour, aggression, and background characteristics, between autumn 2005 and spring 2006. Responses were anonymous. Adult respondents were paid €15 for filling out the questionnaire, minors €10.

Migration status was measured by country of birth of the head of the household of the respondent (Stronks et al. 2009), and was coded as The Netherlands, Turkey, Morocco, Surinam or the Netherlands Antilles. For further analyses, these were categorised as ‘labour immigrants’ (Turkey/Morocco, *N* = 99) and ‘immigrants from former colonies’ (Surinamese/Antilleans, *N* = 148). The head of the household was defined as the main breadwinner. Table 1 presents the characteristics of the different immigrant groups.

**Table 1:** Characteristics of the sample by immigrant group; the Netherlands, 2005/2006

	Dutch	Labour immigrants	Immigrants from former colonies	*p*
*N*	2,735	99	148	
Age in years (mean, SD)	30.56 (6.20)	30.44 (5.72)	30.29 (6.51)	0.87^#^
Sex (% female)	51.9	40.4	54.1	0.09^$^
Education level (% high)^a^	31.0	37.0	50.0	<0.001^$^

*p* *p* value for differences between groups, *SD* standard deviation, *ns* not statistically significant

^#^ *F* test

^$^Chi-square test

^a^High education level is: completion or current education at the level of first or second stage of tertiary education (levels 5–6 of the International Standard Classification of Education)

Questions on health behaviours, and social and demographic background were derived from routine Dutch health-behaviour monitoring. These questions have been standardised internationally (see Table 2). Regarding health behaviours, they covered the core themes in Dutch health promotion policies, i.e. physical activity, smoking, alcohol, nutrition, safe sex, substance use, and sleep behaviour. Where applicable, core indicators for a behaviour were taken, most noticeably for nutrition. For that we took having breakfast and consumption of fruit and vegetables as relatively easily measurable and valid indicators of overall nutrition (Cornelisse-Vermaat and van den Brink 2007). Delinquency was measured through questions on vandalism, violence and crime against property in the past year, on a 5-point scale ranging from never to three times or more from the short version of the International Self-Reported Delinquency study (ISRD) (Enzmann et al. 2010; Junger-Tas et al. 1994). Aggression was measured by the Physical Aggression and Verbal Aggression scales of the Aggression Questionnaire (AQ) (Buss and Perry 1992), which have been found to be reliable and valid for the Dutch population (Meesters et al. 1996). In our sample, Cronbach’s alphas were 0.66 and 0.64, respectively.

**Table 2:** Measures of health-related behaviours, delinquency, and aggression: item and core reference, way of measurement, label, and range of values

Variable (references)	Item	Operationalisation	Label	Range
Alcohol use (Monshouwer 2008; Reijneveld et al. 2003; Pronk et al. 2004)	How many days a week do you drink alcohol? How many glasses or cans of alcohol do you consume on a day you drink?	Total number of days a week	Alcohol day	≥0
		Total number of glasses a day	Alcohol glass	0–7
		Heavy drinker, on the basis of the numbers of glasses and days	Alcohol normative	0–4
Unsafe sex (de Graaf et al. 2008)	In the last six months, how often have you used a condom in new sexual contacts to avoid sexually transmitted diseases (such as AIDS, Chlamydia and gonorrhoea)?	Condom use in new sexual contacts in last 6 months (1 = always, 5 = never)	Unsafe sex	1–5
Physical activity (Wendel-Vos et al. 2003)	How many days and how much time a day during the last week were you active in the following activities: Light physical activity: From home to school/work: public transport, car or scooter At school/work: reading, writing Domestic: cooking, doing the dishes Sports: <5 METS (adolescents ≤18), <4 METS (adults >18) Moderate physical activity: From home to school/work: cycling At school/work: walking stairs, construction work, digging Domestic: ironing Leisure time: walking, cycling Sports: 5–8 METS (adolescents), 4–6.5 METS (adults) Vigorous physical activity: From home to school/work: walking At school/work: walking or work in which heavy things have to be carried Domestic: cleaning the floor, carrying heavy groceries Leisure time: working in the garden, DIY Sports: >8 METS (adolescents) >6.5 METS (adults)	Total time (in half hours) for: 1. Light physical activity 2. Moderate physical activity 3. Vigorous physical activity	1. Exercise light 2. Exercise moderate 3. Exercise vigorous	≥0 ≥0 ≥0
Breakfast (Cornelisse-Vermaat and van den Brink 2007)	How many days a week do you have breakfast?	Total no. of days a week	Breakfast	0–7
Fruit (Cornelisse-Vermaat and van den Brink 2007)	How many days a week do you eat fruit? On the days you eat fruit, how many portions of fruit do you eat?	Total number of days a week × number of portions/day	Fruit	≥0
Vegetables (Cornelisse-Vermaat and van den Brink 2007)	How many days a week do you eat vegetables? On the days you eat vegetables, how many spoonfuls do you eat?	Total number of days a week × portion size/day	Vegetables	≥0
Sleeping behaviour (Meijer et al. 2001)	At what time do you turn off the light? At what time do you wake up?	Total hours sleep a day	Sleep	≥0
Smoking (Reijneveld 1998b, 2002; Crone et al. 2003)	Adults: what do you smoke and how many? 1. Number of cigarettes a day 2. Number of cigars a week (1 cigar = 4 cigarettes) 3. Number of packs pipe tobacco/week (pack = 50 cigarettes) Adolescents: how many cigarettes do you smoke a week?	Total no. of cigarettes a day	Smoking	≥0
Delinquency (Junger-Tas et al. 1994)	International self-reported delinquency study (ISRD; short version)	Number of offences against people and property	Delinquency in last year Delinquency in past	0–11
Aggression (Buss and Perry 1992; Meesters et al. 1996)	Aggression questionnaire	Items on aggressive acts	Physical aggression Verbal aggression	0–60
Drug use (Monshouwer 2008; Reijneveld et al. 2003)	Have you used the following drugs? 1. Cannabis (hashish, marihuana or ‘wiet’) 2. Amphetamine (pep, speed) 3. XTC (ecstasy, MDMA) 4. LSD 5. Cocaine (also crack\boiled coke\freebase) 6. Hallucinogenic mushrooms (paddos or magic mushrooms) 7. Heroin (horse, smack or brown) 8. Methadone Never, in the last 4 weeks, in the last 12 months, not in the last 4 weeks, more than 12 months ago	Never used drugs/soft drugs (1) in last 12 months, not last 4 weeks/soft drugs (1) in last 4 weeks/hard drugs (2–8) in last 12 months, not last 4 weeks/hard drugs (2–8) in last 4 weeks	Drug abuse	0–4
Red light walking/car (Feenstra et al. 2002)	How often have you ignored a red light in the last month: 1. Driving a car or riding a motorbike 2. Walking	Total times you ignored a red light in the last month 1. Driving car/motorbike 2. Walking	1. Red light car 2. Red light	≥0 ≥0

*METS* metabolic equivalent of task

### Analysis

First, we compared the prevalence rates of health behaviours between the two immigrant groups and the indigenous Dutch group using logistic regression analyses, adjusted for differences in age and sex. For these analyses, health behaviours were dichotomised as either meeting the Dutch recommendations on healthy life styles or not.

Second, we assessed differences in the clustering of health behaviours, rule-breaking behaviour and aggression between immigrant groups and the indigenous Dutch group by assessing whether a previously fitted model for the entire group of adult respondents (van Nieuwenhuijzen et al. 2009) also fitted the three separate groups. We did this by using Confirmatory Factor Analyses (CFA) in a structural equation modelling framework to be able to scale skewed categorical variables such as the questions on health behaviours that we used (with a relatively number of respondents reporting ‘no’). CFA provides loadings which not only indicate the strengths of the relationships between behaviours, but also the way in which they each belong to a cluster. A higher factor loading indicates that the cluster is defined more by that behaviour than another behaviour with a lower factor loading. A factor loading is significant if the estimated value divided by the standard error >1.96. A good model fit is indicated by the Comparative Fit Index (CFI) and Tucker-Lewis Index (TLI) (both ≥0.95), and the Root Mean Square Error of Approximation (RMSEA) (≤0.05).

This second step showed that clustering differed by migration group. Therefore, in a third step we built new models for the separate groups by conducting Exploratory Factor Analyses (EFA) and CFA. We had no explicit hypotheses about the structure of the model and thus first conducted an EFA with two, three and four factors. Next, we conducted a CFA to confirm these results, to prevent capitalization on chance. Finally, we performed multi-group analysis which compares the model fit and factor loadings of the two separate immigrant groups.

Missing data on behavioural outcomes were imputed from covariates, using a maximum likelihood approach assuming missing at random (MAR) and pair-wise present data. Missing data concerned <5% of cases for all outcomes. Differences in prevalence rates by migration status were computed using SPSS 16 (http:\\www.spss.com). The imputations and all other analyses were conducted using MPlus (http:\\www.statmodel.com).

## Results

Table 3 shows that prevalence rates of use of alcohol were lower among all immigrant groups compared to the indigenous Dutch group, and skipping breakfast occurred more frequently among immigrants from former colonies.

**Table 3:** Risky behaviours (i.e. not meeting recommendations for healthy lifestyles) of immigrant groups vs. indigenous Dutch group, adjusted for age and sex: prevalence rates (P), odds ratios (OR) and 95% confidence intervals (CI), The Netherlands, 2005/2006

	Dutch (*N* = 2,735)	Labour immigrants (*N* = 99)			Immigrants from former colonies (*N* = 148)			Overall *p* value^a^
	P (%)	P (%)	OR	95% CI	P (%)	OR	95% CI	
Alcohol	27.3	16.2	0.48**	(0.28–0.82)	18.9	0.63*	(0.41–0.96)	0.003
Smoking	30.7	27.3	0.81	(0.52–1.28)	24.3	0.73	(0.50–1.08)	0.20
Drugs	8.1	7.1	0.83	(0.38–1.83)	7.4	0.91	(0.48–1.73)	0.87
Sex	4.2	5.1	1.17	(0.46–2.94)	5.4	1.30	(0.62–2.73)	0.76
Delinquency	3.4	4.0	1.14	(0.41–3.21)	1.4	0.39	(0.09–1.60)	0.41
Skipping breakfast	26.7	35.4	1.44	(0.94–2.19)	38.5	1.75**	(1.24–2.47)	0.002
Not enough fruit	82.3	75.8	0.65	(0.40–1.04)	85.8	1.30	(0.81–2.08)	0.10
Not enough vegetables	87.6	82.7	0.65	(0.39–1.15)	87.2	0.95	(0.58–1.56)	0.35
Physical inactivity	5.4	4.1	0.71	(0.26–1.96)	6.8	1.31	(0.67–2.55)	0.57
Dangerous traffic behaviour	38.4	34.3	0.80	(0.52–1.22)	33.1	0.80	(0.56–1.14)	0.28

Dutch recommendations for a healthy lifestyle are: a maximum of 2 (women) or 3 (men) glasses of alcohol on a maximum of 5 days a week, no smoking, no drug use, using condoms for new sexual contacts, no delinquent behaviour during last year, having breakfast at least 5 days a week, 2 pieces of fruit a day at least 5 days a week, 4 spoonfuls of vegetables at least 5 days a week, a minimum of 30 min of physical exertion on at least 5 days a week, and not ignoring red lights while walking or driving

* *p* < 0.05, derived from a Wald test, ** *p* < 0.01, derived from a Wald test

^a^ *p* values are derived from log likelihood ratio tests

**Fig. 1:**
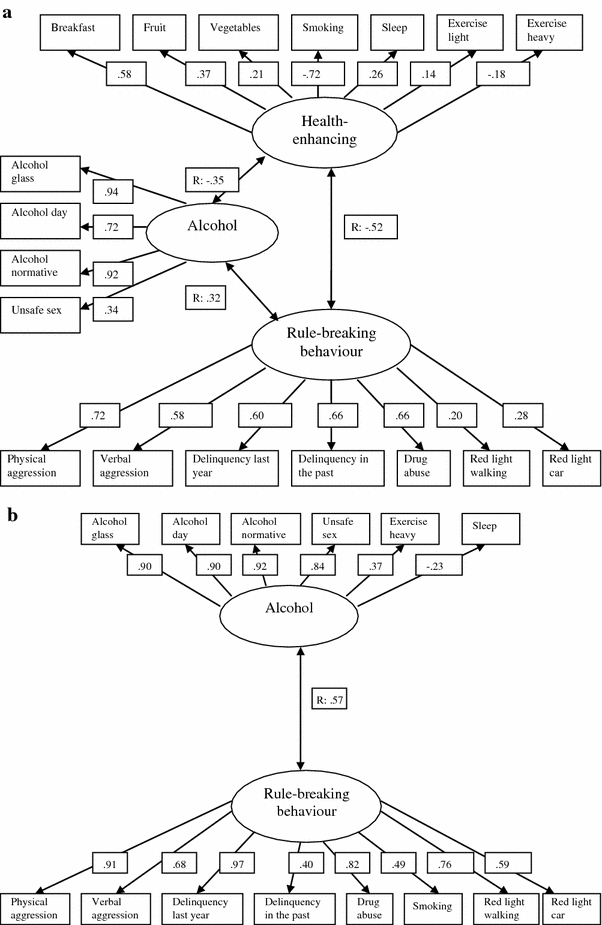

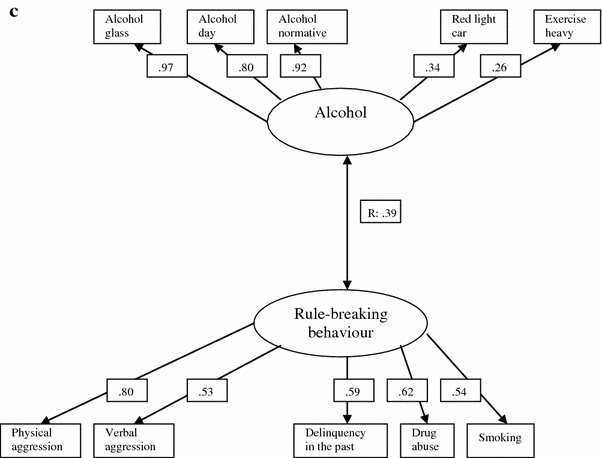
Factor structure and loadings per cluster of risky behaviours, and correlation coefficients between clusters for **a** the indigenous Dutch group in the Netherlands (2005/2006), **b** labour immigrants in the Netherlands (2005/2006), **c** immigrants from the former colonies of the Netherlands (2005/2006). Note: only variables with statistically significant factor loadings have been included

Next, we assessed whether the previously fitted model of the clustering of health behaviours, rule-breaking behaviour and aggression in the entire group of respondents (van Nieuwenhuijzen et al. 2009) applied to each of the three migration groups. That model comprised three clusters: (1) alcohol, which comprises drinking alcohol and unsafe sex, (2) health-enhancing behaviour, which comprises healthy nutritional habits (having breakfast, sufficient fruit and vegetables), enough sleep and physical exercise and no smoking, and (3) rule-breaking behaviour, which comprises delinquency during the last year and in the past, physical and verbal aggression, drug abuse and unsafe traffic behaviour (ignoring red lights when walking or driving a car). For the indigenous Dutch group, this model showed a good fit (χ^2^ = 918.38, df = 104, *p* < 0.001, CFI = 0.95, TLI = 0.95, RMSEA = 0.05; see Fig. 1a). Although there were three individual clusters, the health and rule-breaking behaviour clusters correlated relatively strongly (*r* = −0.52), and the alcohol cluster correlated moderately both with the health cluster (*r* = −0.35) and with the rule-breaking behaviour cluster (*r* = 0.32).

Next, we examined whether this general model for the total population fitted the two immigrant groups as well. This proved not to be the case. Therefore, we built a new model for each of the immigrant groups, using EFA. For labour immigrants we found a reasonably fitting model comprising two clusters: (1) alcohol/unsafe sex/vigorous physical activity/no sleep and (2) rule-breaking behaviour/smoking (χ^2^ = 44.23, df = 24 *p* = 0.01, CFI = 0.92, TLI = 0.94, RMSEA = 0.09), see Fig. 1b. Although we found two separate clusters, they correlated relatively strongly (*r* = 0.57). For immigrants from former colonies we found a well-fitting model comprising two clusters: (1) alcohol/unsafe traffic/vigorous physical activity and (2) rule-breaking behaviour/smoking (χ^2^ = 64.58, df = 49 *p* = 0.07, CFI = 0.95, TLI = 0.95, RMSEA = 0.05), see Fig. 1c. Although we found two separate clusters, they correlated moderately (*r* = 0.39).

Results for the labour immigrant and former colony groups seemed to be rather similar. To assess whether this was indeed the case, we performed a multi-group analysis. This yielded no well-fitting joint model, neither when factor loadings were constrained nor when loadings were free (CFI = 0.87, TLI = 0.89, RMSEA = 0.09), indicating that the clustering among these two immigrant groups did indeed differ.

In the resulting clusters, factor loadings regarding the alcohol components of the cluster alcohol tended to be rather similar across the various groups, with much more variation regarding the composition and loadings of the other variables. Regarding the cluster rule-breaking behaviour, aggression and delinquency contributed across all groups, with a variety of other variables. Among the labour immigrant group, the loadings of current behaviours were relatively stronger than delinquency in the past, compared to the other groups. Moreover, correlations between clusters also differed across groups, being relatively highest among the indigenous groups (between the clusters rule-breaking behaviour and health) and among labour immigrants (between the clusters rule-breaking behaviour and alcohol).

## Discussion

This first comparative study on differences in the clustering of health behaviours, rule-breaking behaviour, and aggression by migration status shows differences in this clustering by migration status. Among the indigenous Dutch group we found three clusters (alcohol, health behaviour, and rule-breaking behaviour), whereas we found two clusters for each of the two immigrant groups (rule-breaking behaviour/smoking and one that differed by group but consistently comprised alcohol and vigorous physical activity). In all three groups the alcohol variables had the highest loadings regarding the cluster alcohol, implying that this could be a particular target for prevention regarding that cluster.

A rule-breaking behaviour cluster was found in all three groups, but in the immigrant groups smoking was part of it, whereas, in the indigenous group smoking clustered with health-enhancing behaviours. An explanation may be that smoking is a more deviant behaviour in immigrant groups, compared to the indigenous Dutch. This may be a relict of the rather low smoking rates among some first-generation immigrant groups (Hosper et al. 2007; Nierkens et al. 2006; Reijneveld 1998a) but apparently not among all. The positioning of smoking among immigrants in a more deviant cluster may be translated into the design of preventive interventions. The same applies to the fact that past delinquency contributes relatively less to this cluster among the labour immigrant groups than among the other groups.

In neither immigrant group do health-enhancing behaviours form a separate health cluster. Some of them are part of the other two clusters, and nutrition is completely unrelated to the other clusters. This may be interpreted as immigrants in this sample being similar to the indigenous Dutch group regarding the clustering of risky behaviours, but not regarding health-enhancing behaviours, and specifically not regarding ‘healthy’ nutrition. Apparently immigrants have their own patterns of nutrition, which is also shown by their lower breakfast rates. Nutrition is a rather strong measure of culture, maybe even its cornerstone (Dubowitz et al. 2007; Nicolaou et al. 2006), and it also differentiates between different immigrant groups. The varying composition of the clusters and of their correlations may also be interpreted as immigrant groups differing in regard to the mutual relationships of health and risk behaviours, not only from the indigenous Dutch, but also from each other. This also implies that an integrated approach may be of use in all three groups, but that they should be targeted at somewhat different combinations of behaviours.

The immigrant groups in this study were combined on the basis of their migration history. However, within the group of labour immigrants the Turks and Moroccans have different eating patterns, and similarly within the group of immigrants from former colonies, the Surinamese and Antilleans have also different eating patterns (Nicolaou et al. 2006). This deviant position of nutrition may even be the reason why no health cluster was found for the immigrant groups, the other behaviours in that cluster have too little coherence to confirm the existence of a cluster without nutrition. Regarding this, it could also be questioned whether the process of migration, i.e. permanently moving to a different country, is the key issue in the differences in clustering, or ethnicity and culture in a broader sense. The latter seems likely, but this certainly deserves further study.

Looking at the components and loadings of behaviours in the various clusters, some behaviours were not in the same clusters for the two immigrant groups, and even when they were, some factor loadings differed between the groups indicating that behaviours contribute to clusters in a different way. This implies that the clustering of health behaviours, rule-breaking behaviour and aggression in each of the two immigrant groups not only differs from the indigenous Dutch group but also from the other immigrant group. For instance, among all immigrant groups heavy exercise is in the same cluster as alcohol, whereas among the indigenous Dutch group it is in the cluster of health-enhancing behaviours. This may be due to the fact that physical exercise has a different meaning in the immigrant groups under study, implying that they tend to do less leisure physical activity (de Munter et al. 2010). Evidently, this should have consequences for preventive interventions.

### Strengths and limitations

Important strengths of this study concern the inclusion of a broad range of health behaviours, and rule-breaking behaviour and aggression, and the inclusion of two well-defined immigrant groups, labour immigrants and immigrants from former colonies. Other strengths of this study are the method of data collection which can be expected to limit information bias including the effects of social desirability, and the response rates which are even slightly higher among immigrants, probably reflecting the quality of the fieldwork. In addition, missing values were imputed and the skewedness of the data is properly accounted for, which provides much more precise results.

A potential limitation concerns the restriction of our sample to immigrants who were able to fill out internet-based questionnaires. They were, thus, relatively acculturated to Western society, which is reflected by their prevalence rates of risky behaviours being rather similar to that of the indigenous Dutch population and their educational level being relatively high. However, even among these relatively acculturated immigrants, clustering differed from the indigenous Dutch group. Among less acculturated immigrants, differences can be expected to be even larger, given the strong relationship between acculturation and change in health behaviours towards those of the majority population among various immigrant groups and for various health behaviours (Hunt et al. 2004; Salant and Lauderdale 2003). Moreover, access to the internet has been shown to be similar among indigenous and immigrant people in the period concerned (2005: 83 and 80%, respectively; 2009: 93 and 96%, respectively (Sleijpen 2010). Second, we could not examine sex differences because of the small sample sizes, although the occurrence of risky behaviours is known to differ between men and women (Hosper et al. 2007; Nierkens et al. 2006; Yang et al. 2009), and it is likely that this applies to immigrant groups in a different way. Third, we used country of birth to measure ethnicity, which may have led to the missing of ethnic differences within a specific country, as well as cultural differences. However, this has been shown to be stable and mostly valid (Stronks et al. 2009). A final limitation is the relatively small sample sizes of the various groups, but even then we found differences in clustering between migration groups which shows that these differences are relatively large.

### Implications

Our findings may have major implications for the development of prevention programmes. First, the clustering as found implies that several separate health behaviours may be addressed simultaneously in integrated prevention programmes for all groups, instead of only targeting separate health behaviours. For some behaviours, immigrant groups and indigenous people may be addressed simultaneously, as our results show that the clustering of some health behaviours is similar among immigrants and the indigenous Dutch population. This in particular concerns use of alcohol and rule-breaking behaviour like aggression, delinquency, and drug use. The added value of such an integrated prevention approach probably applies to immigrants from non-industrialised countries in various industrialised countries, but this has to be confirmed in future studies, particularly for immigrants who are not literate in the dominant language of the country they have migrated to.

With regard to the clustering of having breakfast and consumption of fruit and vegetables, we not only found differences between the indigenous Dutch group and immigrant groups, but also within the immigrant groups. This may be interpreted as eating patterns varying per immigrant group. Specific immigrant groups should, therefore, be addressed via separate approaches.

Our findings should be confirmed using larger immigrant samples that also comprise immigrants who cannot read Dutch and are likely to be least acculturated (Hunt et al. 2004; Salant and Lauderdale 2003) to examine whether a similar clustering can be found among them. Larger differences in clustering are likely to occur among them, given the differences in prevalence rates of the various behaviours. We found such differences regarding use of alcohol, which has been found in other studies as well (Hosper et al. 2007; Nierkens et al. 2006; Reijneveld 1998a). Previous studies among the entire range of immigrants in the Netherlands have shown similar differences in prevalence rates for other health behaviours, which are likely to lead to larger differences in clustering as well. Differences in clustering should also be studied for separate groups within the categories that we used in our current study, for example instead of ‘labour immigrants’, ‘immigrants from Turkey’ and ‘immigrants from Morocco’ separately, or even ‘immigrants of Berber ethnicity from Morocco’, as some authors have done (Stronks et al. 2009). Our findings show that this can support new approaches to establish better health behaviours among immigrant groups, but also help them to maintain their favourable position regarding several behaviours. To realise these new integrated approaches in prevention, additional information is also needed on the influential shared determinants of the clusters as identified per group.

We conclude that:

- Among indigenous adults, health and risk behaviours co-occur in three clusters, alcohol, health behaviour and rule-breaking behaviour
- Among acculturated immigrants from non-industrialised countries two clusters were found, alcohol and rule-breaking behaviour/smoking
- Nutritional patterns do not cluster with other health behaviours among immigrant groups whereas they do among indigenous adults
- Findings support a more integrated approach to promote healthier lifestyles among indigenous adults and immigrant adults who are able to read Dutch, but these approaches should differ regarding, for example, nutrition and physical activity, and they should probably also differ per separate immigrant group.
- For the design of effective integrated interventions, additional information is needed on shared determinants for the various clusters of health and risk behaviours, and potential differences regarding this by sex.
